# Bridging the Gap: Immune Checkpoint Inhibitor as an Option in the Management of Advanced and Recurrent Cervical Cancer in Sub-Saharan Africa

**DOI:** 10.7759/cureus.69136

**Published:** 2024-09-10

**Authors:** Izuchukwu F Okpalanwaka, Francis I Anazodo, Zimuzor L Chike-Aliozor, Chika Ekweozor, Kossy M Ochie, Onyeka F Oboh, Faustina C Okonkwo, Munachiso F Njoku

**Affiliations:** 1 Department of Immunotherapeutics and Biotechnology, Texas Tech University Health Sciences Center, Abilene, USA; 2 Department of Pharmaceutical and Medicinal Chemistry, University of Nigeria, Nsukka, NGA; 3 Department of Biochemistry and Molecular Biology, Augusta University Medical College of Georgia, Augusta, USA; 4 Department of Global Health and Health Security, Taipei Medical University, Taipei, TWN; 5 Department of Clinical Pharmacy and Pharmacy Management, University of Nigeria, Nsukka, NGA; 6 Department of Clinical Pharmacy and Pharmacy Management, Nnamdi Azikiwe University, Awka, NGA; 7 Department of Public Health, School of Nursing and Healthcare Leadership, University of Bradford, Bradford, GBR; 8 Department of Microbiology, University of Nigeria, Nsukka, NGA; 9 Department of Medicine, University of Nigeria, Enugu, NGA

**Keywords:** cancer mortality, cervical cancer, immune checkpoint inhibitors, sub-saharan africa, tumor mutational burden

## Abstract

Cervical cancer remains a leading cause of cancer-related mortality in women in low and middle-income countries despite efforts to improve prevention and standard-of-care interventions. Sub-Saharan Africa (SSA) leads the numbers for global cervical cancer incidence and mortality, with the majority of the incidence diagnosed in the late stage of the malignancy. Although the global cervical cancer death rate has been on the decline for the last two decades owing to advancements in screening and treatment options, the mortality rate in SSA has not declined very much. Chemotherapy has been the treatment of choice for cervical cancer in SSA without meeting the expected survival outcomes in these patients, with the majority having advanced diseases at diagnosis. Immune checkpoint inhibitors have recently shown clinical promise in improving the survival of patients with advanced cervical cancer and have been integrated into the treatment guidelines in most high-income countries, which have helped further reduce the mortality rate of cervical cancer. However, many SSA countries are yet to fully benefit from using immune checkpoint inhibitors in cervical cancer. In this review, we discuss the challenges hindering the effective use of immune checkpoint inhibitors for advanced cervical cancer in Africa and possible solutions.

## Introduction and background

Cervical cancer remains a major health problem worldwide [1]. It is the fourth most common cancer in women and was estimated to have an incidence of over 1.9 million and over 600,000 deaths globally in 2023 [2]. Although largely preventable through screening programs and vaccination, incidence, and mortality are disproportionately distributed globally. The highest rates are in low- and middle-income countries in sub-Saharan Africa (SSA), Southeast Asia, and Central America. Indeed, these regions accounted for most of the 341,831 deaths in 2020 [3], primarily as a result of differences in access to cervical cancer screening services, human papillomavirus (HPV) vaccination, and social and economic determinants [4].

According to a systematic review, in 28 African countries, only 14% of women between the ages of 30 and 49 had ever had a cervical cancer screening as of 2020, with significant regional and country-level differences [5]. In a different survey involving 23 African nations, the majority of healthcare professionals acknowledged lacking sufficient training and awareness on the HPV vaccine, while only 37.4% of them said they had access to it [6].

HPV, precisely types 16 and 18, is responsible for the majority of precancerous cervical lesions and cervical cancers, accounting for over 70% of all invasive cervical cancer cases [7]. The World Health Organization established a global plan in 2020 to eradicate cervical cancer as a threat to public health by 2030 [3]. This strategy included the 90-70-90 targets, which stipulate that 90% of girls must be vaccinated against HPV by the age of 15, 70% of women must have a high-performance test screening by the age of 35, and again by the age of 45, 90% of pre-cancerous lesions must be treated, and cases of invasive cancer must be managed [7,8]. Currently, there are about six HPV vaccines available worldwide by 2023 [9,10]. Recent data have shown that HPV vaccination significantly reduced cervical cancer cases [11,12]. This underscores the need for more vaccination campaigns with broader and far-reaching coverage.

Low-cure systemic medicines are the cornerstone of treatment for distant metastatic cancer, whereas total hysterectomy combined pelvic lymphadenectomy plus concurrent chemotherapy and radiation therapy is the primary treatment strategy for early-stage and locally invasive disease [13]. Due to the need for more sophisticated treatments with higher cure rates, immunotherapy (currently the most promising anticancer strategy) was assessed for use in cervical cancer. As cancer immunotherapy research advances and gains traction, immune checkpoint inhibitors (ICIs) have emerged as a significant new treatment option for patients with recurring and metastatic cervical malignancies. In many wealthy nations, cervical cancer treatment guidelines now include the use of ICIs [14,15].

The importance of immune checkpoints in SSA cannot be overstated, as up to 80% of cervical cancers in SSA are diagnosed in the late stages of the disease, especially considering the clinical success of ICIs in patients with advanced disease [16-18]. Also, as Africa strives to increase cervical screening per WHO targets, it is expected that more cases of invasive cervical cancer will be detected, mainly in the population that had not previously undergone screening. As a result, cancer management techniques must be implemented and expanded, and any challenges must be identified and resolved. This review aims to identify and discuss the challenges of SSA pertaining to utilizing the immune checkpoint inhibitor option in the management of cervical cancer.

## Review

Epidemiology of cervical cancer in SSA

**Figure 1:**
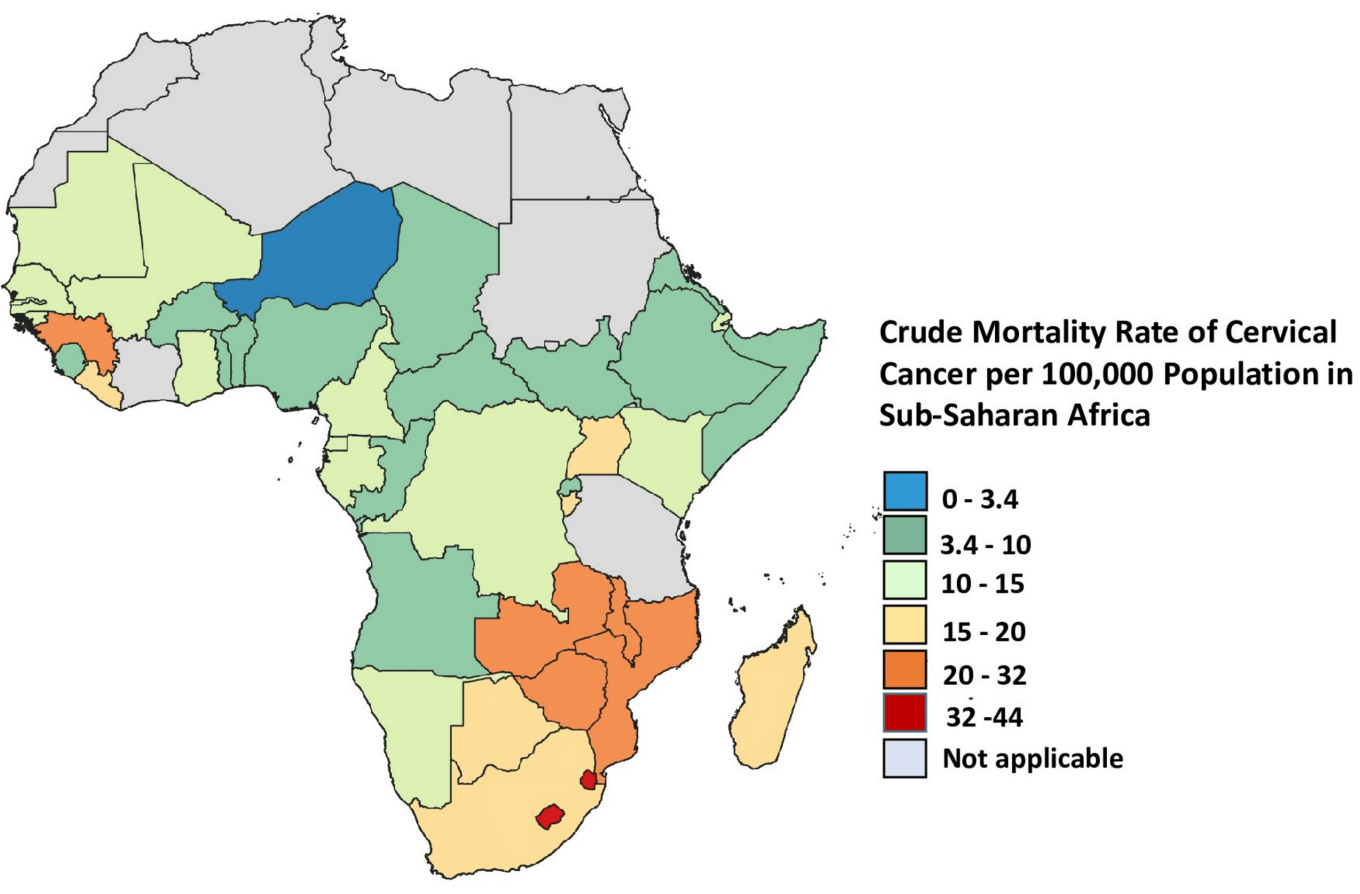
Cervical cancer mortality rate in sub-Saharan Africa. The crude mortality rate among women of all ages who were diagnosed with cervical cancer in sub-Saharan African countries in 2022. This is an original work of the authors graphed from data collected by the International Cancer Control Partnership (ICCP) [23].

According to recent African cancer data, SSA has the highest prevalence of cervical cancer [19], with greater incidence and fatality rates in eastern Africa (with Malawi having the highest burden), southern Africa, and middle Africa [20]. In contrast to the 27 to 30 cases per 100,000 women in the central and western regions and the 40 to 43 cases per 100,000 women in the eastern and southern African regions, North Africa recorded the fewest cases in 2018. There were approximately seven cases per 100,000 women in North Africa. The majority of North Africa's low incidence rate of cervical cancer is due to advancements in screening technology [19]. Furthermore, in northern Africa, sexual and reproductive health behaviors may be influenced by sociocultural and religious norms, which could reduce the incidence of cervical cancer. Over the past four years, the HPV vaccination campaigns in a few regions of eastern, central, and western Africa have marginally reduced the incidence rate [11]. The cervical cancer mortality rate is higher in southern Africa, as shown in Figure 1. Also, mortality rates are declining only in southern Africa. Over the past four years, the mortality rates in every other African region have increased, except for northern Africa, where the incidence rate is low but the fatality rate is high [19]. In 2022, comparable patterns were observed, with the highest incidence and fatality rates found in eastern Africa [11,20,21]. Furthermore, the highest age-standardized rates (ASR) of incidence and mortality are found in SSA nations [22].

Compared to high-income nations, where the five-year survival is above 50%, women with cervical cancer in SSA countries had a five-year survival rate of only 33% [24-26]. According to a study comprising eight SSA countries (the Gambia, Kenya, Malawi, Seychelles, South Africa, Uganda, and Zimbabwe), there has been an alarming rise in the prevalence of cervical cancer in seven of the eight countries. Nonetheless, throughout 25 years, eight eastern and southern African countries, including Malawi, South Africa, and Kenya, saw rising incidence trends, according to an African study [27].

**Figure 2:**
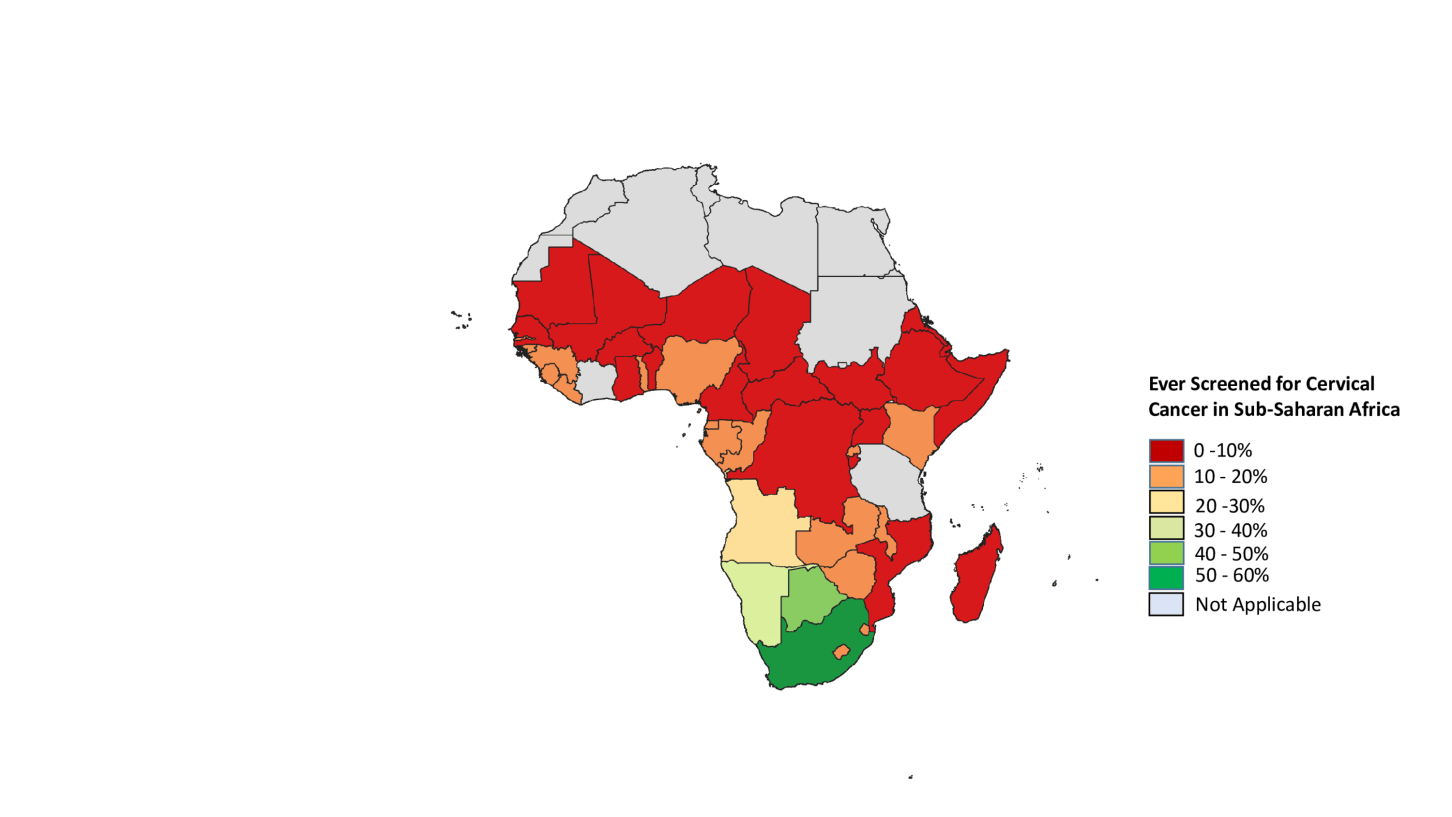
Cervical cancer cancer screening rate in sub-Saharan Africa. This shows the percentage of women screened for cervical cancer at least once in their lifetime in sub-Saharan Africa as of 2019. This figure represents an original work of the authors graphed from data collected by the International Cancer Control Partnership (ICCP) [23].

In SSA, the coverage rate for cervical cancer screening was 10% [28]. Because primary (HPV vaccine) and secondary (screening) prevention measures are so successful, cervical cancer is considered nearly avoidable. These laws haven't, however, been enacted consistently between or within nations. National HPV vaccination programs were present in less than 30% of low- and middle-income countries (LMICs) but in more than 80% of high-income countries [29]. With the lowest prevalence among women in SSA [30], just 44% of women in LMICs had ever had a cervical screening, compared to 60% or more in high-income nations [20]. As Figure 2 shows, screening coverage in SSA is still low despite efforts by governmental agencies to screen more women. 

Cervical screening has been unsuccessful in these areas due to governmental, sociocultural, and financial barriers [31,32]. Some of the systemic challenges that this region faces, which are reflective of their socioeconomic status, include the high cost of Pap smear tests, limited access to service providers, and lengthy wait periods [33]. Cancer patients in SSA are frequently diagnosed after the disease has advanced. Inadequate funding for cancer prevention and early detection is also a contributing factor [34].

If preventative actions like HPV vaccination and screening are not increased, trends in these groups will probably continue to worsen over the coming decades. The global population's aging and growth will also increase the absolute number of cases [35]. Ensuring the implementation of resource-dependent screening and vaccination programs is crucial in these places to improve the situation [36].

Concept of immune checkpoint inhibition

The inhibition of immune checkpoints is an essential aspect of immunotherapy. Immune checkpoints exist to prevent excessive T cell responses on antigen activation, thereby avoiding autoimmunity and supporting peripheral tolerance [37]. An efficient T cell activation requires additional signals (a costimulatory signal and cytokine action) in addition to the first signal provided by the interaction between the T cell receptor (TCR) and an antigen-bounded major histocompatibility complex (MHC) [37,38]. However, when a T cell gets activated, there are also coinhibitory signals delivered by surface proteins to help checkmate the extent of response and maintain immune homeostasis [39]. Programmed cell death protein 1 (PD-1) and cytotoxic T-lymphocyte antigen-4 (CTLA-4) are two well-studied T cell receptors that negatively regulate T cell activation through interactions with their ligands while the antigen-presenting cells (APCs) express the required ligands for interaction with these inhibitory molecules. CD80 and CD86, present on APCs, can bind to the inhibitory CTLA-4 [40]. Similarly, the PD-1 ligands, PD-L1 and PD-L2, are on APC membranes [41]. Most tumor microenvironments (TME) have high expression of immune checkpoint receptors and their ligands. Precisely, tumors can mimic APCs by expressing and upregulating the ligands to T cell coinhibitory receptors [42]. The implication is that the cytotoxic effect of CD8+ T cells on the tumors is abolished. The characteristic upregulation of ligands by tumors exists in various malignancies, including cervical carcinoma, where PD-L1 was reportedly expressed in up to 50% of the cases [42,43]. Like cancer cells, pathogens can harness immune checkpoints to reduce the extent of the host’s antigen-specific immune responses against them [44]. Evans et al. reported that HPV-positive cervical cancers displayed more significant levels of many immune checkpoint proteins compared to cervical cancers that were HPV-negative [45]. Immune checkpoint blockade therapies mainly consist of monoclonal antibodies used to hamper the coinhibitory molecules or their ligands and improve the function of antitumor T-lymphocytes [38]. A good number of clinical trials exploring the use of immune checkpoint inhibitors against cervical cancer are currently ongoing, with some also involving a combination therapy approach, as seen for other carcinomas [46]. An inhibitor of PD-1, pembrolizumab, already has FDA approval for the treatment of advanced, metastasized, or recurrent cervical cancer [47]. We now look briefly at the signaling mechanisms associated with some immune checkpoint (IC) receptors, focusing on the PD-1/PD-L1 axis and CTLA-4.

PD-1

PD-L1 and PD-L2 expressed on either tumor cells or APCs interact with PD-1 on T cells and initiate the resulting signaling. In some cases, the interaction can occur on the same cell in a cis-fashion, or tumors could generate the ligands as exosomes [48]. Phosphorylation of PD-1 on an ITIM/ITSM motif present in its cytoplasmic domain recruits a protein tyrosine phosphatase SHP2 that can inhibit both TCR signaling and the downstream events following T cell activation [41,48]. A contrasting idea also exists with data showing that the dephosphorylation of the costimulatory CD28 is more preferred to the inhibition of the TCR by the PD-1-recruited SHP2 [49]. A disruption in Ca2+ flux in T cells as the levels of PD-1 expression increased is also reported [50]. This model of PD-1 inhibitory effect of T cells showed that higher numbers of engaged TCRs were needed to start a Ca2+ flux as PD-1 signaling increased. The PD-1-mediated pathways reduce T-cell expansion, cause less cytotoxicity against tumors, and decrease cytokine response [51]. Targeting PD-L1 is another option when an immune checkpoint inhibitor (ICI) is not designed to antagonize PD-1. PD-L1 can internalize itself in cycles and then recycle to the cell surface or degrade in lysosomes. Pathways or molecules preventing PD-L1 ubiquitination and degradation can stabilize its expression in carcinomas, making the ligand available to suppress T cell activation through engagement with PD-1. Palmitoylation of PD-L1 or the expression of COP9 signalosome 5 in cancer cells has been reviewed as some of these pathways [48,52].

CTLA-4

CTLA-4 inhibitory action on T cells is multi-dimensional. Following T-cell activation, CTLA-4 translocates from its intracellular position to the cell surface, leading to engagement with the costimulatory B7 ligands (CD80 and CD86) on an APC [40]. CD28, the costimulatory molecule on T cells for CD80 and CD86, is outcompeted by CTLA-4 based on affinity with outcomes of attenuated effects of PI3K and AKT, which are mediators of CD28 signaling [53]. Furthermore, CTLA-4 can affect TCR-induced RAS activation when CTLA-4’s YVTM motif is phosphorylated to recruit SHP2 as the mediator [48,54]. There are cell extrinsic modulations of T cell activity by CTLA-4. One way is through regulatory T cells (Tregs), which also express CTLA-4. CTLA-4 on Tregs may reduce available B7 ligands for CD28-induced stimulation of proximal effector T cells. In addition, CTLA-4 expression can promote transendocytosis of B7 ligands from APCs to prevent interaction with CD28 [53]. 

Clinical experience using ICIs in cervical cancer

The mechanism of PD-1-mediated T-cell suppression is the most well-researched when using ICI to treat cervical cancer. As seen in Table 1, ICIs have been studied in numerous clinical trials as a single drug or in combination for cervical cancer. It is expected that the clinical use of ICIs in cervical malignancies will increase in the coming years.

As seen in Table 1, ICIs have been studied in numerous clinical trials as a single drug or in combination for cervical cancer. It is expected that the clinical use of ICIs in cervical malignancies will increase in the coming years. 

**Table 1:** Immune checkpoint inhibitors used in the clinical trials of cervical cancer. AE: adverse event, DCR: disease control rate, DOR: duration of response, DFS: disease-free survival, DLT: dose-limiting toxicity, ICI: immune checkpoint inhibitor, MTD: maximum tolerated dose, NCT: National Clinical Trial, ORR: overall response rate, CRR: cumulative response rate, OS: overall survival, PFS: progression-free survival, R2PD: recommended phase 2 dose, CD8: cluster of differentiation 8, FOXP3: forkhead box P3, VB10.16: vaccine for human papillomavirus type 16.

NCT number	Drug name	Phase	Primary outcome measure	Status	References
NCT05588219	Tislelizumab	2	Tumor regression	Recruiting	[55]
NCT05824468	Zimberelimab	2	MTD, ORR, RP2D	Not yet recruiting	[56]
NCT05824494	Cadonilimab	2	ORR	Not yet recruiting	[57]
NCT03144466	Pembrolizumab with radiotherapy	1	MTD, efficacy	Terminated	[58]
NCT04256213	Nivolumab + ipilimumab	Pilot study (N/A)	CD8+/FOXP3+ relative change of lymphocytes	Active (not recruiting)	[59]
NCT03755739	Pembrolizumab	2/3	OS, CRR	Recruiting	[60]
NCT03841110	Nivolumab + pembrolizumab	1	MTD	Completed	[61]
NCT04652076	Pembrolizumab+ chemotherapy	1/2	DLT occurrence, ORR	Recruiting	[62]
NCT06140589	Cadolinimab	Not applicable	ORR, PFS, DFS, OS	Not yet recruiting	[63]
NCT05310383	Tislelizumab	2	ORR	Unknown	[14]
NCT06232083	Cardunizumab	1/2	PFS	Recruiting	[64]
NCT05187338	Pembrolizumab + durvalumab + ipilimumab	1/2	DCR, PFS, DOR	Recruiting	[65]
NCT03556839	Atezolizumab	3	PFS, OS	Active, not recruiting	[66,67]
NCT05799469	Envafolimab+ chemoradiotherapy	2	PFS	Not yet recruiting	[57]
NCT05492123	Nivolumab+ iplimumab	2	3-year PFS	Recruiting	[68]
NCT04157985	PD-1/PD-L1 inhibitors	3	PFS, time to next treatment	Recruiting	[69]
NCT03612791	Atezolizumab + radiotherapy	2	PFS	Active, not recruiting	[70]
NCT04405349	Atezolizumab + VB10.16 vaccine	2	AE, ORR	Completed	[71]
NCT04800978	Durvalumab + BVAC-C vaccine	2	DLTs, PFS	Not yet recruiting	[72]
NCT03228667	ICIs + immunotherapy	2	ORR	Active, not yet recruiting	[73]
NCT03527264	Nivolumab + radiation	2	PFS	Terminated	[74]
NCT04300647	Tiragolumab + ICI	2	ORR	Active, not yet recruiting	[75]
NCT03444376	Pembrolizumab + vaccination	1/2	DLT, ORR	Completed	[76]
NCT03786081	Tisotumab vedotin	1/2	DLT, ORR	Active, not yet recruiting	[77]
NCT04802876	Spartalizumab + tislelizumab	2	ORR	Recruiting	[78]
NCT02628067 (KEYNOTE 158)	Pembrolizumab	2	ORR	Recruiting	[79]
NCT03635567 (KEYNOTE 826)	Pembrolizumab+ chemotherapy	3	PFS, safety, efficacy	Active, not yet recruiting	[80]
NCT02488759 (CHECKMATE 358)	Nivolumab monotherapy	1/2	ORR	N/A	[81]

Clinical trials such as KEYNOTE-158, KEYNOTE-826, and CHECKMATE-358 explored the use of anti-PD1 antibodies like pembrolizumab and nivolumab in cervical cancers that were either advanced, PD-L1 positive, or previously treated with chemotherapy [79-81]. The FDA approved pembrolizumab in treating metastatic or recurrent cervical cancer as a result of these trials [47,82]. The National Comprehensive Cancer Network (NCCN) also suggests nivolumab as a component of second-line therapy for cervical cancer [83]. Antibodies targeting PD-L1 have also been developed and are being tested. Durvalumab and atezolizumab are two such antibodies [46]. The desire to prevent extreme adverse effects, improve patient response, and promote overall survival implies that new molecules, even with the same targets, are continuously being developed for cervical cancer treatment. Ipilimumab was developed as an antagonist to CTLA-4, and the FDA has approved it for treating metastatic melanoma [84]. In the CHECKMATE-358 trial, ipilimumab was used in a combination therapy approach against cervical cancer alongside nivolumab with outcomes of durable clinical activity [85]. Balstilimab plus zalifrelimab are an anti-PD-1/anti-CTLA-4 combination, and both inhibitors yielded longer response duration and manageable safety profiles compared to their use as a monotherapy [86]. Evidence for the expression of PD-L1 in cases of cervical carcinoma in SSA and the high mortality from the disease in the same region supports the case for the incorporation of ICI use in the treatment regimens for cervical cancer in the continent [43,46].

Challenges hindering the usage of immune checkpoint inhibitors in SSA

The Lancet Oncology Commission for Sub-Saharan Africa has projected that by 2040, the number of cancer-related deaths in SSA will have doubled, from about 550,000 to over 1 million annually [87,88]. This calls for strategic approaches aimed at reducing the increasing cancer mortality. ICIs have improved the treatment landscape of advanced tumors. A combination of the ICI, pembrolizumab with chemotherapy improved the progression-free survival (PFS) in patients with advanced cervical cancer compared to those taking chemotherapy with placebo (10.4 months vs. 8.2 months) as seen in the KEYNOTE trial [89,90]. FDA approval of pembrolizumab for advanced cervical cancer underlies the key role immune checkpoint inhibitors can play in reducing the high mortality rate associated with gynecological cancers. The following issues need to be addressed to effectively use ICIs in treating cervical cancer in SSA.

Tumor Mutational Burden (TMB) Genomic Sequencing

Tumor mutational burden represents the number of somatic mutations observed per megabase in a tumor DNA [91-93]. In recent times, TMB has become an essential biomarker predicting the sensitivity of immune checkpoint inhibitors as cancer treatments. Tumors with high mutations (TMB-H) have higher chances of recognition by the immune system. This is because neoantigens found in high mutational tumors are more recognizable by the immune cells. High mutational burden correlates positively with high microsatellite instability (MSI-H) [92]. TMB-H and MSI-H are tumor phenotypes showing evidence as predictive biomarkers of ICI sensitivity [94]. The FDA in 2020 approved ICI drug pembrolizumab for any solid tumor with tumor mutational burden-high [≥10 mutations/megabase (mut/Mb)] [95]. This further shows the importance of robust TMB testing in predicting patients' suitability as candidates for ICI therapy. Tumor analysis from ovarian cancer patients showed that TMB-H was associated with better survival and better immune cell tumor infiltration [96]. TMB-H cervical cell carcinoma development is potentially adversely impacted by the activities of anti-tumor immune cells [97]. Patients with MSI-H tumors in endometrial cancer have shown significant therapeutic benefits from dostarlimab in combination with carboplatin-paclitaxel, according to the results of a clinical trial [98]. This, therefore, supports the idea that patients with gynecological tumors showing TMB-H or MSI-H will benefit from ICI therapy.

It has been established in non-small cell lung cancer (NSCLC) that patients of African ancestry show high TMB, suggesting likely benefits for ICI therapy [99]. Numerous efforts have been geared toward elucidating the TMB outlook in many gynecological tumors, including cervical cancer. Genomic testing in SSA will help establish adequate literature necessary to understand different mutations involved in driving several gynecological tumors and help determine patients who are candidates for immunotherapies. Over 30 genes have been investigated worldwide for their potential to influence the risk of cervical cancer [100,101]. Despite the high death rate from cervical cancer in SSA, less than 30% of these genes have been examined in this group. The epidemiological roles of several genes in cervical cancer have been investigated [102]. These genes include CCR2, Casp8, p53, FASL, HLA, IL10, TGF-beta, and TNF-alpha. Only 12% of cancer immunotherapy centers use genomic sequencing to measure tumor mutational burden as a predictive biomarker of immunotherapy success, according to a recent study carried out in 28 SSA nations [103]. Lack of infrastructure and poor logistics are part of the issues making genomic sequencing difficult and uninteresting to medical practitioners in SSA [104].

Bridging the gap in tumor genomic sequencing in African cervical cancer patients is very important if the current high mortality rate observed in this category of patients is to be reduced. Studies of the tumor mutational burden of SSA cervical cancer patients and comparing it to those of other races can help improve our understanding of predicted ICI sensitivity in SSA cervical patients since clinical trial data of ICI use in cervical cancer in other locations are available. One primary solution to the issues of poor genomic sequencing in SSA is boosting public-private partnership (PPP) in genomic sequencing initiatives. For example, less than 10 SSA countries have been able to conduct robust cervical cancer epidemiological studies [102,105]. Many efficient genomic centers in SSA are championed by for-profit or non-profit private organizations [104]. While these institutions have made tremendous progress in changing the genomic landscape of tumor genomic sequencing in SSA, much work is still needed as Africa contributes to less than 2% of the human genome database [106]. Also, genomic sequencing costs are not yet affordable for most cervical patients in SSA. With PPP, the governments of SSA countries can make commitments that reduce the cost individual cervical cancer patients bear when ordering a genomic study, as they also have to bear the cost of treatment for their cancer [107].

PD-L1 Testing in SSA

Since the last decade, PD-L1 has become a vital biomarker predicting the effectiveness of ICIs in treating cancers [90]. PD-L1 overexpression in cervical cancer has been established with suggestions it is linked to high expressions of cancer stem cell markers [108]. High levels of PD-L1 expression in many malignancies have been linked to a bad prognosis [109], but they have also proven helpful in assisting physicians in determining whether patients with cancer are likely to benefit from ICI therapy, which has grown to be a significant treatment option for advanced cancer patients. For patients whose tumors have a PD-L1 Combined Positive Score (CPS) of ≥1, the FDA approved pembrolizumab, an anti-PD-1 antibody, in 2021 in conjunction with chemotherapy to treat metastatic cervical cancer [110]. To understand the genomic and molecular landscape of cancer, many studies have sought to evaluate racial variations in the expression of PD-L1 in different tumors, which have yielded mixed results so far [109,111]. More studies are needed to fully understand how PD-L1 is influenced by race for each cancer type.

Despite the role of Africa in human evolution and the rich genetic diversity of the zone, Africa remains behind in genetic databases and studies [112]. A study found that less than 50% of the participating clinics in SSA conduct PD-L1 testing before immunotherapy is used in cancer treatment [103]. A global survey of pathologists doing immunohistochemistry testing of PD-L1 found that Africa had a longer turnaround time (TNT), representing the time from testing to when the result becomes available. The survey also found that reporting the results is less standardized in Africa compared to the other continents that participated [113]. Longer TNT is one such problem that can affect the confidence of healthcare providers managing cervical cancer patients in ordering PD-L1 testing, as quick decision-making might be necessary when dealing with an advanced form of the disease. Embracing the benefits of next-generation sequencing (NGS) is one way SSA can improve the current less impressive genomic and diagnostic testing situation. PPPs remain the most viable option in increasing the number of diagnostic centers in SSA capable of performing PD-L1 testing. This will lead to shorter TNT.

Lack of Updated Treatment Guidelines in SSA

A 2019 symposium organized by the Cervical Cancer Research Network (CCRN) assessed the current management of cervical cancer in eleven SSA countries and found that platinum- and taxol-based chemotherapy are the most used therapy for metastatic or recurrent cervical cancer while radiation therapy or chemoradiation was majorly used in palliative care for cases where the cancer was deemed incurable in the participating SSA countries [114]. These strategies are not in line with the latest management used in many high-income countries for the management of advanced disease that has metastasized to multiple sites. In the United States, it is recommended that for those who have recurrent disease or have progressed on chemotherapy, a combination strategy involving bevacizumab should be used [115]. The suggested combinations include cisplatin and paclitaxel with bevacizumab [115]. However, the recent success of the KEYNOTE trial inspired FDA approval of the anti-PD-1 ICI, pembrolizumab to be combined with platinum-based chemotherapy with or without bevacizumab for metastatic cancer with good expression of PDL1 [90]. In the United Kingdom and the European Union, pembrolizumab has been approved and recommended for use, similar to the FDA approval for cervical cancer [116]. Pembrolizumab should be added to chemotherapy with or without bevacizumab in patients with cervical cancer that is chronic, recurring, or metastatic, according to the most recent practice guideline published by the Korean Society of Gynecologic Oncology [117]. To reduce the increasing mortality of cervical cancer in SSA, there is a need to develop efficient clinical guidelines that adopt the latest clinical evidence in the management of advanced cervical cancer. In the event that developing country-specific guidelines proves difficult, regional-based guidelines can be mapped out based on collaboration among multiple SSA countries, which can help speed up the process.

High Cost of ICIs

ICI therapy has advanced the field of clinical oncology, and many positive patient outcomes have been reported. However, the cost of ICIs remains high and out of reach for many patients from LMICs. The average cost of one dose of pembrolizumab is reported to be $8762 [118]. Many late-stage cervical cancer patients would typically require more than one dose. The cost of a single dose of pembrolizumab is more than the yearly minimum wage reported in most SSA countries, making it very challenging for many cervical cancer patients to afford such therapy out of pocket. Yet over 80% of the population of most SSA countries get access to medicine by paying out-of-pocket [119]. In contrast, only about 13% of the population under 65 in the USA is without health insurance [120,121]. Health insurance coverage is an effective way to reduce the financial burden of a costly medical need. Although many SSA countries have introduced universal health insurance (UHI) programs with the hope of bringing cheap and affordable health care to many poor people who lack access to essential health, these schemes have yet to meet the expected need [122]. Challenges like poor funding, administrative bottlenecks, and inadequate primary care facilities are among the major stumbling blocks towards achieving an effective public health coverage scheme that meets the health needs of low-income people who struggle to afford good health care [123,124]. To ensure access to ICIs for cervical cancer patients, the governments of SSA countries should invest in subsidizing the therapy cost so patients can afford it. Information sharing and proper patient referral systems should be built to ensure that patients with advanced cervical cancer are referred to hospitals with adequate facilities to manage their conditions properly.

## Conclusions

Since the clinical benefits of immune checkpoint inhibitors (ICIs) in improving patient outcomes in advanced cervical cancer were established, many countries, especially in high-income areas of the world, have outlined guidelines for the effective use of ICI therapy in cervical cancer patients with PD-L1 expression. In recent times, sub-Saharan Africa (SSA) has experienced an increased incidence of cervical cancer with an increasing mortality rate. To benefit from the clinical success of ICIs in cervical cancer, SSA countries need to address the challenges hindering the use of ICIs. To effectively address these issues, public-private partnerships will play a vital role in achieving that. The responsibility of establishing efficient genomic and biomarker testing facilities should not be left in the hands of private firms, as it has recently been in Africa. The governments of the countries of SSA need to provide an enabling environment with facilities aimed at improving logistics and other social amenities needed for biomedical research to thrive. The governments can also coordinate partnerships with countries that have successfully used immunotherapies in cervical cancer to help adopt a framework that can be implemented in Africa. Without an updated guideline that gives authority to ICIs as a beneficial therapy in advanced cervical cancer, getting all health institutions in SSA countries in line with what is obtainable in higher economy countries will be challenging. The health ministries of the various countries of SSA, through their affiliated medical associations and societies, should be tasked with reviewing the current guidelines and ensuring that the current practice is similar to what is currently done in countries where there has been a decrease in cervical cancer mortality in recent years. Finally, more research and clinical trials involving ICIs are encouraged in the SSA countries to help shine a light on how cervical cancer patients with advanced disease respond to ICI therapy compared to the currently-used chemotherapy. This will help decide the best and most efficient way to use these therapies for the patient’s benefit.
